# Ethno-psychopharmacological aspects of treatment response in patients with delusional syndrome: A systematic review

**DOI:** 10.1192/j.eurpsy.2021.427

**Published:** 2021-08-13

**Authors:** A. Guàrdia, A. González-Rodríguez, M.V. Seeman, A. Alvarez Pedrero, M. Betriu, J.A. Monreal, D. Palao Vidal, J. Labad

**Affiliations:** 1 Mental Health, Parc Taulí University Hospital. Autonomous University of Barcelona (UAB). I3PT, Sabadell, Spain; 2 Mental Health, Parc Taulí-University Hospital, Sabadell, Spain; 3 Psychiatry, University of Toronto, Toronto, Canada; 4 Mental Health, Parc Tauli-University Hospital, Sabadell (Barcelona), Spain; 5 Mental Health, Hospital of Mataró. Consorci Sanitari del Maresme. CIBERSAM., Mataró, Spain

**Keywords:** Delusional disorder, cultural and ethno-biological factors., potential influences, treatment response

## Abstract

**Introduction:**

Treatment response in schizophrenia can be influenced by cultural and ethno-biological factors. However, in delusional disorder (DD), these potential influences have been poorly investigated.

**Objectives:**

This review aims to synthesize what is known about the influence that cultural and biological factors may have on treatment response in DD.

**Methods:**

A systematic review was performed on PubMed from inception to 2020 in keeping with PRISMA directives. Search terms: [(cultural OR ethnic* OR ethno*) AND (treatment OR therap* OR antipsychotic response) AND (delusional disorder)]. We included all studies whose objective was to explore ethno-psychopharmacological aspects of treatment response in DD.

**Results:**

A total of 182 papers were retrieved. Four studies tested ethno-biological factors and 10 reported cultural aspects of treatment response in DD. 1. Cultural hypothesis: 3 studies reported cultural differences in diagnostic practices; in 2 studies, culturally-determined long durations of untreated psychosis (DUP) and comorbidity with mood disorders was associated with response to both antipsychotics (AP) and antidepressants (AD); 3 studies reported that response and AP dose were similar among cultures and that culturally-sensitive psychotherapy improved adherence; 2 studies showed that, where women had poor access to health care, mortality rates were high. 2. Ethno-biological hypothesis: 1 study reviewed moderators and mediators of ethno-specific treatment response; 1 study presented a culture-bound syndrome (Taijin kyofusho) for which AD were found effective; 2 studies in diverse populations found that DD and schizophrenia were both significantly linked to HLA genes.

**Conclusions:**

The sociodemographic profile of DD is consistent across various cultures and, when treated appropriately, responds, but in an ethno-culturally-specific manner.

**Disclosure:**

No significant relationships.

